# TCOF1 affects Golgi secretory pathway contributing to the angiogenesis in renal cancer

**DOI:** 10.1186/s12964-026-02796-1

**Published:** 2026-03-17

**Authors:** Małgorzata Grzanka, Piotr Popławski, Jacek R. Wiśniewski, Roksana Iwanicka-Nowicka, Helena Kossowska, Marta Koblowska, Beata Rybicka, Alex Białas, Agnieszka Piekiełko-Witkowska

**Affiliations:** 1 Department of Biochemistry and Molecular Biology, Centre of Postgraduate Medical Education, Warsaw, Poland; 2 Department of Proteomics and Signal Transduction, Max Planck Institute of Biochemistry, Am Klopferspitz 18, Martinsried, Planegg, 82152 Germany; 3 Faculty of Biology, Laboratory of Systems Biology, University of Warsaw, Warsaw, Poland; 4 Laboratory for Microarray Analysis, Institute of Biochemistry and Biophysics, Polish Academy of Sciences, Warsaw, Poland

**Keywords:** TCOF1, Treacle, ccRCC, Renal cancer, Nucleoli, Golgi secretory pathway, THBS1, Thrombospondin 1, Angiogenesis, Treacher-Collins syndrome

## Abstract

**Background:**

TCOF1 is a nucleolar protein involved in ribosome biogenesis, DNA damage response, and mitotic regulation. Germline *TCOF1* mutations are associated with Treacher-Collins syndrome, a rare congenital disorder characterized by craniofacial abnormalities. Clear cell renal cell carcinoma (ccRCC), the most prevalent form of kidney cancer, exhibits pronounced nuclear and nucleolar pleomorphism, which correlates with tumour aggressiveness. The ccRCC grading system relies on microscopic evaluation of nuclear and nucleolar features. Here, we hypothesized that TCOF1 contributes to ccRCC tumorigenesis.

**Methods:**

The study involved 200 tissue samples from ccRCC patients, two ccRCC-derived cell lines, and the publicly available cancer datasets. The used techniques included siRNA transfections, proliferation, viability, migration, and adhesion assays, proteomic and transcriptomic analyses, Western blot, real-time PCR, and angiogenesis evaluation using HUVEC cells.

**Results:**

TCOF1 expression was elevated in ccRCC tumours and correlated with higher nucleolar grade and poorer patient survival. TCOF1 depletion altered the expression of multiple genes and proteins involved in the Golgi secretory pathway, including AVL9, GOLGA4, GOPC, RPS6KA5, SCAMP1, SEC24B, and STEAP3. These changes led to enhanced secretion of the anti-angiogenic thrombospondin 1 and suppression of angiogenesis. Furthermore, TCOF1 silencing downregulated several proteins implicated in craniofacial development, such as DCAF7 (aka WDR68), CHUK, APAF1, DICER1, and ETS1.

**Conclusions:**

To our knowledge, this is the first study linking a nucleolar TCOF1 protein to the regulation of cellular secretion. Our findings suggest that elevated TCOF1 expression may disrupt the Golgi secretory pathway, inhibit thrombospondin 1 secretion, and promote angiogenesis in ccRCC. Our study also contributes to the understanding of the molecular consequences of TCOF1 dysfunction in Treacher-Collins syndrome.

**Supplementary Information:**

The online version contains supplementary material available at 10.1186/s12964-026-02796-1.

## Background

TCOF1 (aka treacle) is a nucleolar protein required for rRNA transcription and ribosome biogenesis [1]. It is also involved in ubiquitination, response to the DNA damage, mitotic regulation, and protection of telomere integrity [1]. TCOF1 mutations are associated with Treacher-Collins syndrome, a genetic disorder characterized by craniofacial abnormalities [2] and neural tube closure defects [3]. Several recent studies have also linked TCOF1 to cancer development and progression, highlighting its potential role in oncogenesis [4–9].

Clear cell renal cell cancer (ccRCC) originates from the proximal tubules and is the most common subtype of kidney malignancies. Primary ccRCC is treated mainly by surgical resection which in case of localized tumours leads to favourable prognosis for > 90% of patients. Patients with metastatic ccRCC are offered a wide spectrum of treatments, including inhibitors of tyrosine kinase receptors (TKI), mTOR/AKT kinases or immune control checkpoints. Unfortunately, most of these patients inevitably relapse and further progress with the disease. The prognosis for metastatic ccRCC patients is poor and overall survival ranges from 16 to 50 months, depending on the site of the metastasis [10]. The grade of ccRCC malignancy is estimated based on the pathomorphological analysis of nuclear and nucleolar pleomorphy [10–12].

Since TCOF1 is a nucleolar protein, we hypothesized that it may contribute to the ccRCC pathology. To this end, we performed comprehensive analyses of the of TCOF1 in ccRCC. We found that TCOF1 expression is upregulated and correlates with nucleolar grade of ccRCC tumors. Surprisingly, we also found that TCOF1 regulates the expression of multiple genes involved in the Golgi secretory pathway, contributing to the secretion of the angiogenic thrombospondin 1 (THBS1). Here, we describe a novel tumour-promoting role of TCOF1 as a regulator of cancerous secretion.

## Methods

ccRCC-derived cell lines, 786-O (cat. no. ATCC^®^ CRL­1932™), Caki-1 (cat. no. ATCC^®^ HTB­46™), and HUVEC/TERT2 (cat. no. ATCC^®^ CRL-4053™) were purchased from ATCC and cultured in accordance with manufacturer’s instructions. 786-O cell line is derived from a primary tumour, while Caki-1 is a metastatic cell line. Both cell lines exert pronounced pro-angiogenic effects in tumour xenografts [13–15].

RNA from ccRCC tumor tissue samples and matched-paired non-tumorous control kidney tissues was retrieved from the local RNA and Tissue Bank at the Department of Biochemistry and Molecular Biology of the Centre of Postgraduate Medical Education under approval of the local Bioethical Committee (approval no. 9/2021).

RNA isolated from tissues was reverse transcribed with Transcriptor First Strand cDNA Synthesis Kit (Roche Diagnostics, Mannheim, Germany) using random hexamer primers and anchored oligo(dT) as described previously [16].

Silencing of TCOF1 was achieved using double transfection with siRNA, as this procedure had proven successful in the preliminary experimental setup, resulting in more than 50% silencing of the target gene (Supplementary Figure S1). Briefly, 786-O or Caki-1 cells were seeded and cultured for 24 h with the following transfection using Lipofectamine 2000 (Thermo Fisher Scientific) and 25nM siRNA1 (Silencer^®^ Select cat. no. 4392420, ID S529267) against TCOF1 or negative control siRNA (Silencer™ Negative Control No.1 siRNA, Cat. no. AM4611/AM4635). After 5 h, cells were transfected for the second time with 25nM siRNA2 (Silencer^®^ Select, cat. no. 4392420, ID S529265) against TCOF1 or the negative control siRNA and incubated overnight. Medium was renewed every 24 h.

RNA isolation from cell lines was done using GeneMATRIX Universal RNA/miRNA Purification Kit (EURX, Gdansk, Poland), in accordance with the protocol provided by the manufacturer.

qPCR was done using cDNA synthesized with Revert Aid H Minus First Strand cDNA Synthesis kit (Thermo Fisher Scientific, Inc) and SYBR-Green I Master (Roche Diagnostics, GmbH) as previously described [17]. Sequences of primers are shown in Supplementary Table S1. Gene expression was normalized to RNA18S1, which was stably expressed in cells with silenced TCOF1 expression (Supplementary Figure S2). TCOF1 expression in tissue samples was measured using TaqMan probes (Hs00947135 (TCOF1), Hs03928985_g1(RNA18S1).

Protein isolation was done by 5 min extraction at 98 °C in a buffer consisting of 0.1 M Tris pH 7.5, 2% SDS, 0.1 M DTT (for 786-O cells) or RIPA Lysis and Extraction Buffer (Thermo Fisher Scientific) with 0.5 mM PMSF and protease inhibitor cocktail 0.1 M (for Caki-1 cells) as earlier described [17].

For WB analysis, 30 µg (for TCOF1 and GOPC analysis) or 20 µg (for SCAMP1 analysis) of protein was resolved using SDS-PAGE (10% acrylamide gel) with the following wet transfer onto nitrocellulose (TCOF1 and GOPC analysis) or PVDF (for SCAMP1 analysis). The membranes were blocked o/n in 5% non-fat milk in TBST, washed three times and incubated with primary antibodies: anti-TCOF1 (cat. no. HPA038237, Sigma-Aldrich, St. Louis, MO; diluted 1: 500 in 5% non-fat milk, o/n), anti-GOPC (cat. no. 12163-1-AP, Proteintech, diluted 1:1000 in 5% non-fat milk, 1.5 h) or anti-SCAMP1 (cat. no. 15327-1-AP, Proteintech, diluted 1:2000 in 5% non-fat milk, 1.5 h) and further processed as described [17]. Anti-β-actin antibody (cat. no. NB600-501, Novus Biologicals) was used as a loading control. The exposed membranes were developed using SuperSignal™ West Dura Extended Duration Substrate (cat. no. 34076, ThermoFisher Scientific).

Immunocytochemistry (ICC) analysis: cells were seeded on coverslips, fixed with 4% paraformaldehyde (PFA), and permeabilized with 0.25% Triton X-100. Non-specific binding was blocked with 2% BSA in TBST. Primary antibody incubation was carried out overnight at 4 °C, followed by incubation with secondary antibody for 1 h at room temperature. F-actin was stained using phalloidin-Atto488 (Sigma-Aldrich, St. Louis, MO), and cell nuclei were stained with DAPI. The following antibodies were used in this study: Fibrillarin (B-1): sc-166,001, 1:250 (Santa Cruz Biotechnology, TX, USA), Anti-TCOF1: HPA038237, 1:400 (Sigma-Aldrich, St. Louis, MO, USA), Sec24B (D7D6S): #12,042, 1:100 (CellSignaling, MA, USA) The imaging was performed using a confocal microscope LSM800 AxioObserver.Z1 and Zen 3.7 software (Zeiss, Oberkochen, Germany).

3D holotomographic imaging: 62,500 cells were seeded onto 35 mm dishes. Imaging was performed using a 3D Holo-Tomographic Live Cell Imaging Microscope (Nanolive, Switzerland) and processed with Steve Full software version 1.6.3496.

Proliferation was evaluated using Cell Proliferation ELISA, BrdU (colorimetric) kit (Roche Diagnostics, GmbH), in line with the instructions provided by the manufacturer.

Viability was analysed using Cell Proliferation Kit I (MTT) (Roche Diagnostics, Mannheim, Germany) and CellTiter 96 Aqueous One Solution Cell proliferation Assay (MTS) (Promega, USA) according to the manufacturer’s protocol.

For the wound healing assay, 2.5 × 10^4^ 786-O or 5 × 10^4^ Caki-1 cells were seeded onto a 12-well plate in a complete medium. 48 h after transfection, scratches were made with a sterile 200 µl pipette tip, and the plates were washed to remove detached cells. Wound area was measured using Incucyte^®^ SX1 Live-Cell Analysis System and analysed with ImageJ with wound healing size tool (https://github.com/AlejandraArnedo/Wound-healing-size-tool).

Adhesion-independent growth was evaluated using CytoSelect™ 96-Well Cell Transformation Assay (Soft Agar Colony formation) (Cell Biolabs, Inc. USA) according to the manufacturer’s protocol.

Collection of Conditioned Media (CM): 24 h and 48 h after transfection, cells were rinsed once with PBS and 4 times with DMEM without phenol red, supplemented with GLUTAMAX. Next, DMEM without phenol red, supplemented with GLUTAMAX. After additional 24 h, CM was collected, centrifuged and frozen at -80 °C for further analyses.

THBS1 ELISA was done using Human Thrombospondin 1 ELISA Kit ELH-TSP1-1 (Raybiotech Life, Inc. USA) in line with the manufacturer’s protocol.

Angiogenesis was evaluated using the tube formation assay. To this end, 1 × 10^4^ HUVEC/TERT2 cells were seeded on 96-wells plate covered with Corning^®^ Matrigel^®^ Growth Factor Reduced (GFR) Basement Membrane Matrix, LDEV-free. Before seeding, HUVEC cells were resuspended in CM from 786-O or Caki-1 cells and cultured for 48–78 h. Results were analyzed using Angiogenesis Analyzer for Image J [18].

Microarray analysis was done as previously described [19] using Affymetrix™ HuGene 2.1 ST Array Strips (Affymetrix, Santa Clara, CA, USA). Data were deposited in NCBI GEO (acc. no. GSE299580).

Proteomic analysis: Proteins were extracted with 0.1 M Tris-buffer (pH 7.5) containing 2% (w/v) SDS and 0.1 M DDT at 98 °C for 5 min. The obtained protein extracts were processed by the MED-FASP method as described in [19]. Total protein and total peptide were determined using the tryptophan fluorescence-based assay [20]. Peptides were analyzed by LC-MS/MS using Q-Exactive HF mass spectrometer (Thermo Scientific, Waltham, MA, USA) [21]. Spectra were analyzed using the MaxQuant software. Proteins were quantified using the TPA approach [22]. Statistical analysis was done using Student’s t-test with a cutoff of a 1.5-fold change. The mass spectrometry proteomics data have been deposited to the ProteomeXchange Consortium via the PRIDE [23] partner repository with the dataset identifier PXD027601.

Bioinformatic analysis was done using ENCORI [24] and UALCAN [25]. Data from TCGA KIRC [26] and CPTAC clear cell renal cell carcinoma [27] cohorts were used for the analyses.

Statistical analysis was done with GraphPad Prism 10, using a t-test on data from at least three independent biological experiments. *P* < 0.05 was considered statistically significant.

## Results

### TCOF1 expression is upregulated in renal cancer and correlates with poor survival of patients

**Fig. 1:**
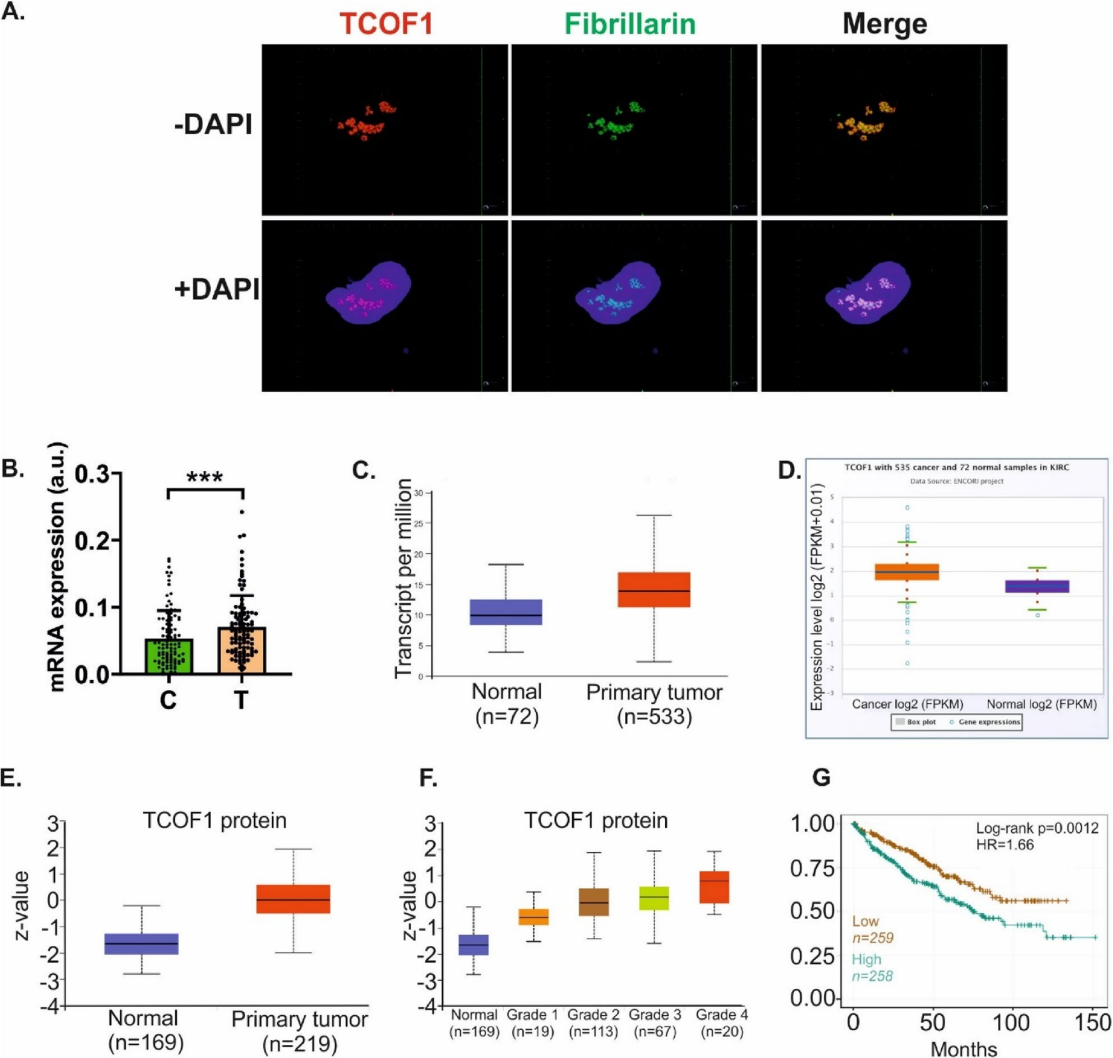
TCOF1 expression is increased in ccRCC tumors. **A** Nucleolar localization of TCOF1 in 786-O cells confirmed by ICC staining. Red: TCOF1, blue: nucleus (DAPI stain), red: fibrillarin (nucleolar marker). 3D images were obtained using Z-stack imaging. **B** Expression of TCOF1 mRNA in matched-paired ccRCC tumours (T: *n* = 100) and non-tumorous kidney samples (C: *n* = 100). The plots show the results of qPCR analysis. Statistical analysis was performed using Wilcoxon signed-rank test. *** *p* < 0.001. **C** TCOF1 mRNA expression in TCGA data (UALCAN platform); *p* = 3.3 × 10^-16; *n* = 72 normal kidney tissues, *n* = 533 primary tumour tissues; **D** TCOF1 mRNA expression in TCGA data (ENCORI platform); *p* = 7.7 × 10^-16, FDR = 5.2 × 10^-15, *n* = 72 normal kidney tissues, *n* = 535 tumour tissues. **E** TCOF1 protein expression in CPTAC data; *p* = 1.1 × 10^-69, *n* = 169 normal kidney tissues, *n* = 219 (primary tumour tissues). **F** TCOF1 protein expression in ccRCC tumors classified according to tumour grade. p-values are given in Supplementary Table S2. **G** Correlation of TCOF1 expression with patients’ survival. Kaplan-Meyer plot was generated using ENCORI platform (KIRC cohort). *N* = 517

Confocal microscopy confirmed that TCOF1 formed foci inside nucleoli of ccRCC cells which confirmed previous observations in myogenic cells [28] (Fig. 1A**).** TCOF1 expression was increased in ccRCC tumors when compared with non-tumorous matched-pairs of kidney samples (Fig. 1B). This was also confirmed by the analysis of publicly available datasets (Fig. 1C, D, E). Moreover, high TCOF1 expression increased with the malignancy grade and correlated with poor survival of patients (Fig. 1F and G).

### TCOF1 silencing affects transcriptome and proteome of ccRCC cells

The correlation of high TCOF1 expression with malignancy grade and poor prognosis for ccRCC patients suggested that it could contribute to the cancerous progression. Therefore, we checked the functioning of ccRCC cells following TCOF1 silencing. To this end, we chose the 786-O cell line, which is the most commonly used and the best characterized in vitro model in RCC research [13]. Efficient siRNA-mediated silencing of TCOF1 was verified using Western blot (Supplementary Figure S3). Surprisingly, TCOF1 depletion did not affect viability, proliferation, migration, or adhesion-independent growth of ccRCC cells (Supplementary Figure S4). Therefore, to get more insight into the TCOF1 function in ccRCC cells, we silenced its expression in 786-O cells with the following microarray and proteomic analysis.

**Fig. 2:**
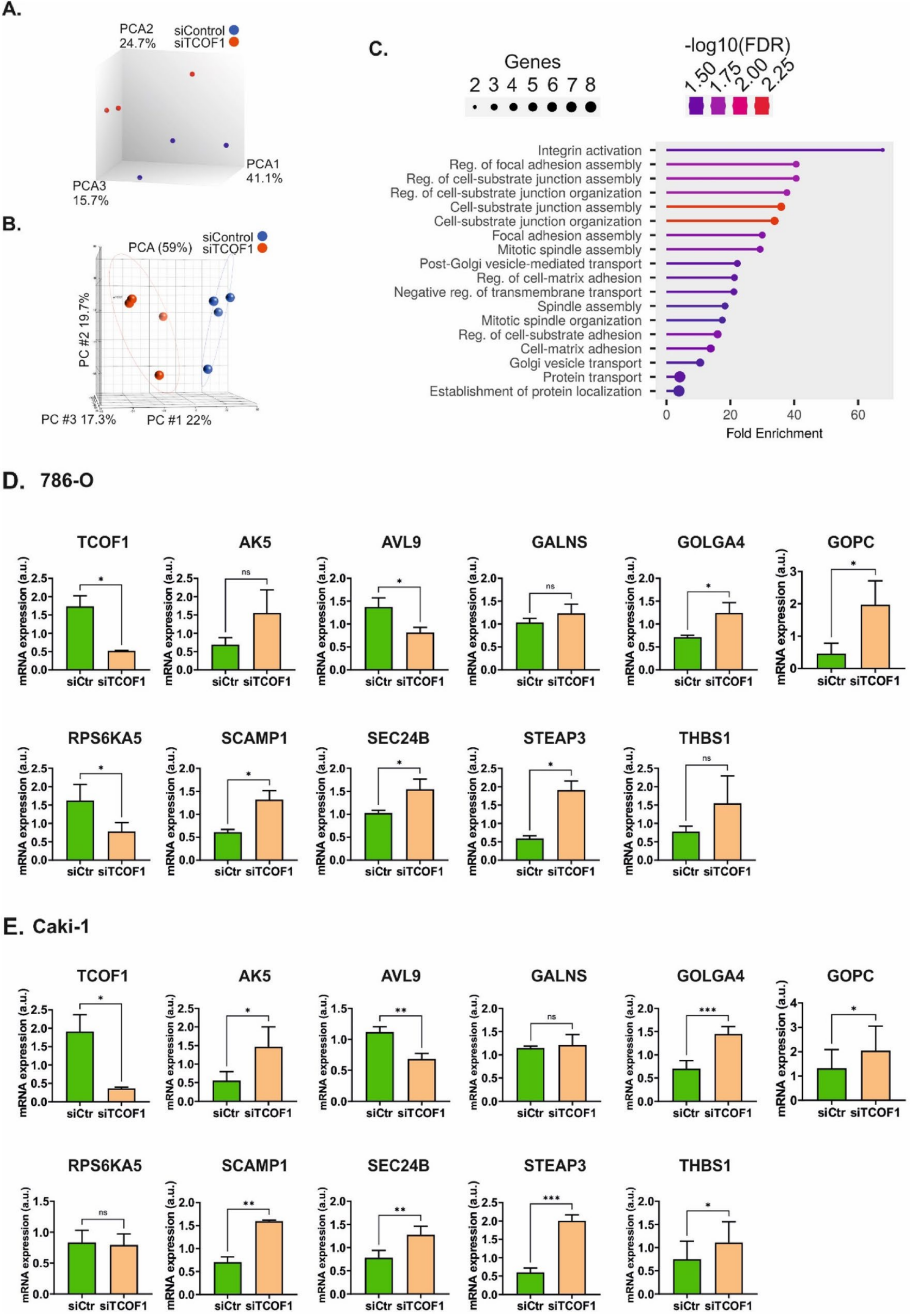
Silencing of TCOF1 affects transcriptome and proteome of ccRCC cells. **A** PCA of microarray analysis of 786-O cells with silenced TCOF1 expression. **B** PCA of the proteomic analysis of 786-O cells with silenced TCOF1 expression when compared with cells transfected with a control non-targeting scrambled oligonucleotide. **C** The most enriched biological processes following TCOF1 silencing in 786-O cells. The plot shows GO analysis of the 26 genes/proteins commonly altered by TCOF1 silencing in microarray and proteomic analysis. **D** qPCR validation of the commonly altered genes in 786-O cells with silenced TCOF1 expression. **E** qPCR validation of the commonly altered genes in Caki-1 cells with silenced TCOF1 expression. Statistical analysis was performed using t-test. *N* = 3 independent biological experiments

PCA of microarray data provided a clear separation of the two groups. TCOF1 silencing resulted in altered expression of 176 genes, including 85 upregulated and 91 downregulated ones (Fig. 2A, Supplementary Table S3). Gene ontology analysis revealed that the most enriched pathways included ECM-receptor interaction, microRNAs in cancer, inositol phosphate metabolism or signalling, as well as focal adhesion. This was reflected by the most enriched cellular components that included protein complexes involved in cell adhesion, lysosomal membrane, or lytic vacuole membrane (Supplementary Figure S5).

Proteomic analysis revealed 451 altered proteins, including 267 upregulated and 184 downregulated ones. The most enriched cellular components included platelet dense granule membrane, mitochondrial matrix, focal adhesion, cell substrate junction, anchoring junction, and Golgi membrane (Fig. 2B, Supplementary Table S4, Supplementary Figure S5).

**Fig. 3:**
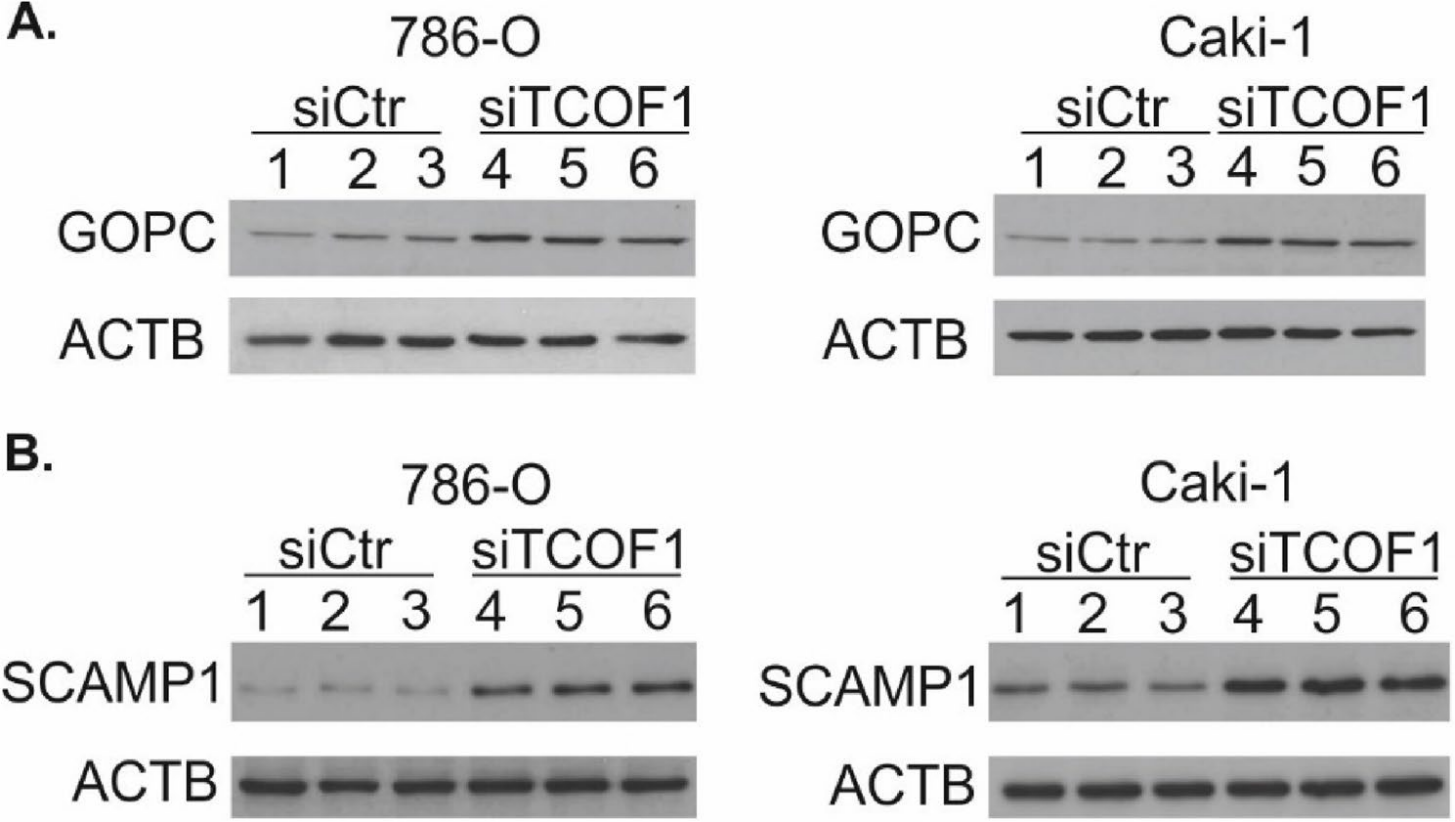
siTCOF1 silencing upregulates expression of proteins involved in Golgi secretory pathway. The images show representative Western blots of GOPC (**A**) and SCAMP1 (**B**) analysed in ccRCC cell lines with silenced (siTCOF1) or not (siCtr) TCOF1 expression. Each path represents protein isolated from independent cell culture bottles

Comparison of the proteomic and microarray data revealed consistently altered expressions of 26 gene-protein pairs (Table 1). Analysis of the biological processes linked with these proteins demonstrated that they were mostly enriched in integrin activation, regulation of focal adhesion assembly, and junction organization of post-Golgi vesicle-mediated transport (Fig. 2C). Notably, the highest number of commonly enriched genes/proteins was involved in protein transport and localization. To validate these data, we performed qPCR analysis in siRNA-transfected 786-O cells as well as in another commonly used ccRCC cell line, Caki-1. qPCR validation confirmed altered expression of genes related to Golgi apparatus, endosome recycling, and vesicle trafficking, including AVL9, GOLGA4, GOPC, RPS6KA5, SCAMP1, SEC24B, and STEAP3 **(**Fig. 2C, E**)**. Moreover, WB analysis confirmed that TCOF1 silencing resulted in upregulation of GOPC and SCAMP1 involved in the Golgi secretory pathway (Fig. 3).

**Table 1:** Genes and protein targets commonly altered by TCOF1 silencing in 786-O cells as revealed by microarray and proteomic analyses. Fold change values (FC) are bolded

Gene names	Protein names	Proteomic analysis		Microarray analysis		
		FC	*P*-value	FC	*P*-value	FDR *P*-val
THBS1	Thrombospondin-1	**10.62**	1.16E-02	**1.58**	3.77E-02	8.01E-01
GOPC	Golgi-associated PDZ and coiled-coil motif-containing protein	**5.89**	1.29E-05	**2.10**	4.99E-06	7.00E-02
AK5	Adenylate kinase isoenzyme 5	**4.97**	2.20E-04	**1.85**	9.70E-03	6.42E-01
GOLGA4	Golgin subfamily A member 4	**4.41**	2.00E-03	**1.55**	2.00E-04	2.03E-01
STEAP3	Metalloreductase STEAP3	**4.04**	3.81E-04	**3.10**	2.50E-05	1.12E-01
SCAMP1	Secretory carrier-associated membrane protein 1	**3.82**	2.55E-03	**2.37**	5.00E-03	5.54E-01
FN1	Fibronectin; Anastellin; Ugl-Y1;Ugl-Y2;Ugl-Y3	**3.41**	4.99E-03	**1.81**	5.00E-04	2.89E-01
LSM14A	Protein LSM14 homolog A	**3.30**	1.02E-03	**2.36**	3.06E-06	7.00E-02
SEC24B	Protein transport protein Sec24B	**3.13**	5.96E-03	**1.91**	4.57E-05	1.30E-01
TLN1	Talin-1	**2.35**	1.42E-04	**1.76**	1.00E-04	1.64E-01
CPNE2	Copine-2	**2.33**	3.06E-03	**1.70**	6.90E-03	5.91E-01
LAMP2	Lysosome-associated membrane glycoprotein 2	**2.16**	1.53E-03	**1.51**	2.00E-04	1.80E-01
GALNS	N-acetylgalactosamine-6-sulfatase	**2.00**	3.35E-02	**1.57**	5.80E-03	5.66E-01
DNMT1	DNA (cytosine-5)-methyltransferase 1	**1.97**	1.34E-02	**1.53**	2.00E-04	2.17E-01
CLASP1	CLIP-associating protein 1	**1.76**	1.57E-03	**1.59**	2.74E-05	1.12E-01
SLC30A1	Zinc transporter 1	**-1.57**	2.20E-02	**-1.70**	2.12E-02	7.34E-01
ACOT13	Acyl-coenzyme A thioesterase 13;Acyl-coenzyme A thioesterase 13, N-terminally processed	**-1.68**	1.67E-02	**-1.66**	5.00E-04	3.00E-01
AVL9	Late secretory pathway protein AVL9 homolog	**-1.82**	7.33E-04	**-1.55**	1.80E-03	4.21E-01
KIF3B	Kinesin-like protein KIF3B; Kinesin-like protein KIF3B, N-terminally processed	**-2.05**	1.48E-02	**-1.65**	2.00E-04	1.80E-01
DBT	Lipoamide acyltransferase component of branched-chain alpha-keto acid dehydrogenase complex, mitochondrial	**-2.20**	2.31E-03	**-1.57**	5.70E-03	5.66E-01
RBBP5	Retinoblastoma-binding protein 5	**-2.22**	2.89E-02	**-1.53**	3.50E-02	7.91E-01
HGD	Homogentisate 1,2-dioxygenase	**-2.27**	4.63E-02	**-1.61**	5.20E-03	5.60E-01
TBC1D13	TBC1 domain family member 13	**-2.30**	4.31E-03	**-1.52**	2.14E-02	7.35E-01
SCML2	Sex comb on midleg-like protein 2	**-2.61**	1.07E-03	**-1.59**	9.10E-03	6.27E-01
TCOF1	Treacle protein	**-2.76**	5.43E-04	**-1.90**	6.00E-04	3.32E-01
FAM73A	Protein FAM73A	**-3.05**	3.16E-02	**-1.97**	3.87E-02	8.05E-01
RPS6KA5	Ribosomal protein S6 kinase alpha-5;Ribosomal protein S6 kinase	**-3.75**	3.51E-03	**-1.65**	2.05E-02	7.31E-01

Altogether, this data suggested that silencing of TCOF1 in ccRCC cells could affect intracellular protein trafficking and secretion.

### THBS1 secretion is altered by TCOF1 silencing

**Fig. 4:**
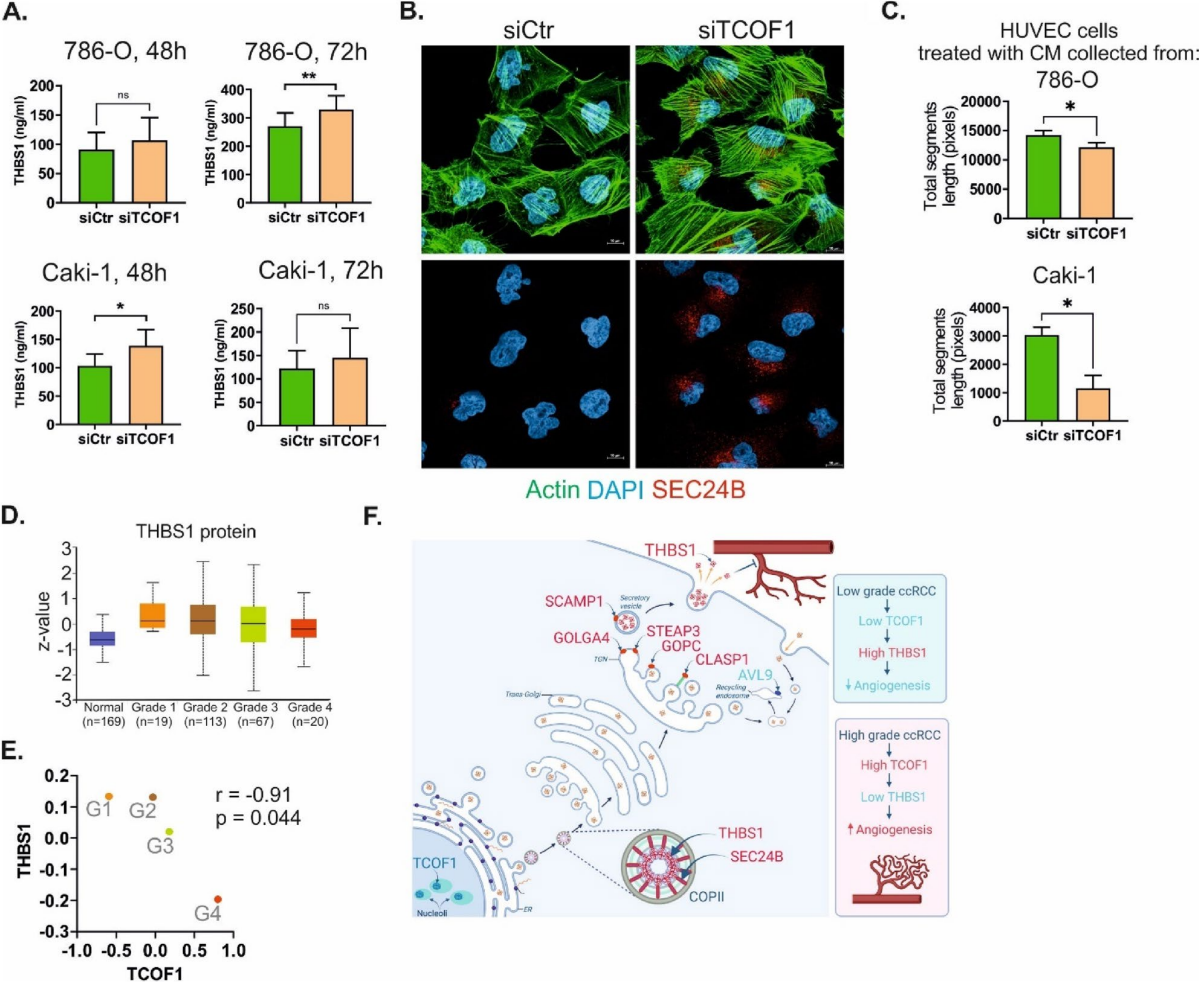
TCOF1 regulates the Golgi secretory pathway and THBS1 secretion in ccRCC cells. **A** Silencing of TCOF1 stimulates secretion of THBS1. The plots show THBS1 concentrations in conditioned media collected from 786-O and Caki-1 cells with silenced TCOF1 expression. *N* = 3 independent biological experiments. Statistical analysis was performed using t-test. * *p* < 0.05, ** *p* < 0.01, ns: not statistically significant. **B** TCOF1 silencing increases expression of SEC24B, a COPII protein. Microscopic images of 786-O cells with silenced (siTCOF1) or not (siCtr) TCOF1 expression are shown. For Caki-1 cells see Supplementary Figure S5. **C** TCOF1 silencing affects angiogenic properties of ccRCC secretome. The plots show total length of segments created by HUVEC cells incubated with conditioned media retrieved from ccRCC cells (786-O, Caki-1) transfected with siRNA against TCOF1 (siTCOF1) or non-targeting scrambled oligonucleotide (siCtr). *N* = 3 independent biological experiments. Statistical analysis was performed using t-test. * *p* < 0.05. Representative microscopic photographs of HUVEC cells are shown in Supplementary Figure S7. **D** THBS1 protein expression decreases with advance of ccRCC tumor grades. *P*-values are given in Supplementary Table S2. **E** THBS1 protein expression negatively correlates with TCOF1 expression across ccRCC tumour grades. The values provided on axes represent median Z-values. The data was retrieved from UALCAN/CPTAC. Pearson r and *p*-values are shown. **F** A model depicting the role of TCOF1 in the Golgi secretory pathway in ccRCC

The omics data revealed that the most upregulated protein in cells with silenced TCOF1 expression was THBS1 (FC 10.62) (Table 1). Interestingly, THBS1 is secreted into the extracellular space and affects tumour microenvironment (TME), including the endothelial cells. To check if indeed TCOF1 silencing affected the amount of the secreted THBS1, we collected conditioned media (CM) from the ccRCC cells with silenced TCOF1 and analysed THBS1 concentrations using ELISA. Indeed, we found elevated THBS1 concentrations in CM collected from ccRCC cells with silenced TCOF1 when compared with cells transfected with non-targeting scrambled siRNA (Fig. 4A).

In kidney cells, THBS1 colocalizes with coatomer protein II (COPII) anterograde vesicle, which facilitates its secretion and anti-angiogenic activity. In contrast, RCC cells retain THBS1 in the endoplasmic reticulum (ER), which facilitates RCC-secretome-induced angiogenesis [29]. SEC24B functions as a cargo-binding component of the COPII vesicle coat [30]. We found that TCOF1 silencing induced expression of SEC24B mRNA (Fig. 2D, E) and protein (Fig. 4B, Supplementary Figure S6). This suggested that in ccRCC cells, high TCOF1 expression may result in low SEC24B expression, preventing its binding with THBS1, contributing to the decreased secretion and blocking its antiangiogenic effect. Indeed, treatment of HUVEC cells with conditioned media collected from the RCC cells with silenced TCOF1 expression attenuated tube formation when compared with RCC cells transfected with non-targeting control siRNA (Fig. 4C). Finally, we checked whether high-grade ccRCC is associated with diminished THBS1 expression. To this end, we took advantage of the same proteomic dataset used for the evaluation of TCOF1 expression (see Fig. 1). Indeed, we found that THBS1 expression decreased as ccRCC grade advanced (Fig. 4D). Moreover, THBS1 protein levels negatively correlated with TCOF1 across ccRCC tumour grades (Fig. 4E).

## Discussion

TCOF1 is a nucleolar protein known for its roles in ribosome biogenesis, nucleolar stress responses, DNA repair, and protection against oxidative and osmotic stress [1, 31]. Our study adds a novel function to this repertoire: the regulation of the Golgi secretory pathway and the secretion of the anti-angiogenic factor thrombospondin 1 (THBS1) (Fig. 4F).

The nucleolus is increasingly recognized as a multifunctional hub regulating gene expression, allelic exclusion, DNA repair, and stress responses [32, 33]. To our knowledge, this is the first study that directly links nucleolar function with the regulation of cellular secretion. However, the mechanistic associations between nuclear morphology and the secretory pathway were suggested previously in basic studies performed in yeast. Specifically, it was demonstrated that mutations of yeast Rab-GTPases YPT6 and YPT32 reduce nucleolar size [34], while Golgi vesicle trafficking is essential for maintaining nuclear shape under membrane proliferation conditions [35]. In our study, TCOF1 silencing induced widespread transcriptional changes in genes involved in the Golgi secretory pathway (Figs. 2 and 3). These included upregulation of GOPC, a PDZ-domain scaffolding protein that regulates secretion of proteins such as insulin [36]. In the canine kidney cells GOPC localizes to the Trans-Golgi Network (TGN) and regulates tight junction structure [37]. We also observed increased expression of STEAP3 (also known as TSAP6), a trans-Golgi protein essential for exosome production and secretion [38]. SCAMP1 (Secretory Carrier Membrane Protein 1), another upregulated gene, encodes a carrier protein involved in vesicle transport and membrane fusion [39]. Expression of CLASP1 (Cytoplasmic Linker Associated Protein 1 or CLIP-Associating Protein 1), a microtubule-associated protein required for the proper post-Golgi trafficking, was also elevated [40, 41]. In contrast, AVL9 (Late Secretory Pathway Protein AVL9 Homolog), a recycling endosome-associated protein, was the only secretory gene downregulated by TCOF1 silencing. In yeast, Avl9p overexpression is toxic and causes post-Golgi secretory defects [42].

Remarkably, in cells with silenced TCOF1 expression, we observed pronounced upregulation of SEC24B, which is involved in the transport of secretory proteins from the endoplasmic reticulum to the Golgi apparatus. Specifically, SEC24B functions as a cargo-binding component of the COPII vesicle coat [30]. In RCC cells, SEC24B is involved in THBS1 secretion. THBS1 is a multifunctional secreted protein that modulates the tumour microenvironment by attenuating angiogenesis and limiting anti-tumour immunity. It also influences cellular responses to radio- and chemotherapy, as well as autophagy and senescence. The anti-angiogenic actions of THBS1 rely on the inhibition of endothelial cell migration and proliferation, as well as the induction of their apoptosis. In vivo, increased THBS1 expression results in diminished vascularity and reduced tumour growth [43]. Given that ccRCC is a highly vascularized tumour and anti-angiogenic therapies are a mainstay of treatment, our finding that TCOF1 silencing enhances SEC24B expression and THBS1 secretion is particularly relevant. Remarkably, it has been demonstrated that, compared with low-grade tumours, high-grade ccRCC tumours exhibit higher angiogenic activity [44]. Taking this into consideration, and given that TCOF1 expression in low-grade ccRCC tumours is diminished compared with high-grade tumours, we hypothesized that ccRCC cell lines with silenced TCOF1 could recapitulate the angiogenic effects observed in low-grade tumours. Conversely, cell lines with unchanged TCOF1 expression could represent the effects seen in high-grade tumours. Indeed, we found that conditioned media from TCOF1-depleted ccRCC cells showed reduced angiogenic activity compared with controls (Fig. 4).

Although our study was focused on renal cancer, our findings also contribute to the understanding of Treacher-Collins syndrome resulting from TCOF1 inactivating mutations. The causal relationship between TCOF1 deficiency and craniofacial malformations has been primarily attributed to impaired oxidative protection, leading to extensive apoptosis of the neural crest precursors, diminishing their number migrating to the first and second pharyngeal arches [45]. Remarkably, our proteomic data (Supplementary Table S4) revealed that silencing of TCOF1 led to a substantial decrease in the expression of numerous genes and proteins involved in craniofacial development, including DCAF7 (aka WDR68) [46], CHUK [44], APAF1 [47], DICER1 [48], and ETS1 [49]. Furthermore, a recent study demonstrated that mutations in SEC24B are associated with congenital vertebral malformations, while SEC24B is essential for neural tube closure [30, 50]. Interestingly, TCOF1 missing mutations have recently been reported in patients with neural tube closure defects [3]. To our knowledge, no studies have yet addressed the proteomic changes in Treacher-Collins syndrome patients; however, it is plausible that such changes may involve altered expression of proteins identified in our study, including SEC24B.

In conclusion, we report a novel, pro-tumorigenic role of TCOF1 in renal cancer. In low-grade tumours, relatively low TCOF1 expression does not interfere with THBS1 secretion, sustaining moderate angiogenic activity. In higher grade tumours, increased TCOF1 expression may modulate Golgi secretory pathway, to reduce THBS1 secretion and facilitate tumorous angiogenesis (Fig. 4F).

## Supplementary Information

Supplementary Material 1: Table S1. Sequences of primers used for qPCR analysis. Table S2. Statistical significance of TCOF1 protein expression changes in ccRCC tumours. Data retrieved from UALCAN/CPTAC. Normal kidney samples: n = 169, Grade 1 ccRCC: n = 19, Grade 2 ccRCC: n=113, Grade 3 ccRCC: n = 67, Grade 4 ccRCC: n= 20. Table S3. The results of microarray analysis of 786-O cells with silenced TCOF1 expression when compared with cells transfected with non-targeting scrambled oligonucleotide. N=3 independent biological experiments. Data were deposited in NCBI GEO (acc. no. GSE299580). Table S4. The results of proteomics analysis of 786-O cells with silenced TCOF1 expression when compared with cells transfected with non-targeting scrambled oligonucleotide. N=4 independent biological experiments. Data have been deposited to the ProteomeXchange Consortium via the PRIDE partner repository with the dataset identifier PXD027601. Figure S1. TCOF1 silencing with double siRNA transfection results in more than 50% suppression of TCOF1 expression. The plot shows TCOF1 mRNA expression (% of control) in 786-O cells transfected with siRNA1, siRNA2 and or sequential transfection by siRNA1 followed by siRNA2. mRNA was isolated 48h after transfection, each bar represents TCOF1 expression in n = 3 wells of cell culture plate. Control: 786-O cells transfected with non-targeting scrambled oligonucleotide. Catalogue numbers and IDs of siRNAs are given in Methods. Figure S2. Stable expression of the RNA8S1 reference gene in cells with silenced TCOF1 expression. N=3 independent biological experiments. Statistical analysis was performed using t-test. *P* < 0.05 was considered statistically significant. Figure S3. Uncropped full scans of WB analysis of TCOF1 silencing in 786-O and Caki-1 cells. Each lane represents analysis of protein extract isolated from independently transfected cell culture flask. TCOF1 migrates at a higher apparent molecular weight than predicted based on its sequence, primarily due to its high content of CK2 phosphorylation sites [51]. Figure S4. TCOF1 silencing does not affect proliferation, viability, migration, and adhesion-independent growth of ccRCC cells. A. Viability. B. Proliferation. C. Migration. D. Adhesion-independent growth. 786-O and Caki-1 cells were transfected with siRNA targeting TCOF1 (siTCOF1) or non-targeting control scrambled oligonucleotide. The number of independent biological experiments: N=3 (panels A, B, C) n=4 (panel D). E. Adhesion -independent growth (n=4 independent biological experiments). RFU=relative fluorescence units. Statistical analysis was performed using paired t-test. Figure S5. GO analysis of microarray and proteomic data from 786-O cells with silenced TCOF1 expression. The graphs shows most enriched cellular components in 786-O cells with silenced TCOF1 expression when compared with cells transfected with control non-targeting scrambled oligonucleotide. A. Data from microarray analysis. B. Data from proteomics analysis. Figure S6. TCOF1 silencing increases expression of SEC24B in Caki-1 cells. TCOF1 was transiently silenced in Caki-1 cells with the following ICC analysis. Scale bar: 10 µm. Blue: nucleus (DAPI), green: GM130 (cis Golgi marker), red: SEC24B. Figure S7. Representative microscopic photographs of HUVEC cells treated with CM from 786-O (A) or Caki-1 (B) cells with silenced (siTCOF1) or not (siCtr) TCOF1 expression. Note diminished length of segments created by HUVEC cells incubated with conditioned media retrieved from ccRCC cells with silenced TCOF1 expression.

## Data Availability

The dataset(s) supporting the conclusions of this article are included within the article and its additional files. Microarray data have been deposited in NCBI GEO (acc. no. GSE299580). The mass spectrometry proteomics data have been deposited to the ProteomeXchange Consortium via the PRIDE (23) partner repository with the dataset identifier PXD027601.

## Abbreviations

ccRCC: clear cell renal cell carcinoma
CLASP1: Cytoplasmic Linker Associated Protein 1 (or CLIP-Associating Protein 1)
CM: Conditioned medium (or conditioned media)
ECM: Extracellular matrix
ELISA: Enzyme-Linked Immunosorbent Assay
ICI: Immune control checkpoints
qPCR: quantitative real-time PCR
TCGA: The Cancer Genome Atlas
TCOF1: Treacle Ribosome Biogenesis Factor 1
THBS1: Thrombospondin 1
TKI: Tyrosine kinase inhibitors
TME: Tumour microenvironment
WB: Western blot
