# Supplementary material for: TCOF1 affects Golgi secretory pathway contributing to the angiogenesis in renal cancer
Source: Cell Commun Signal. 2026 Mar 17;24:249. doi: 10.1186/s12964-026-02796-1 (PMC13107843; doi:10.1186/s12964-026-02796-1)
Supplement: Supplementary file 1 — Supplementary Material 1: Table S1. Sequences of primers used for qPCR analysis. Table S2. Statistical significance of TCOF1 protein expression changes in ccRCC tumours. Data retrieved from UALCAN/CPTAC. Normal kidney samples: n = 169, Grade 1 ccRCC: n = 19, Grade 2 ccRCC: n=113, Grade 3 ccRCC: n = 67, Grade 4 ccRCC: n= 20. Table S3. The results of microarray analysis of 786-O cells with silenced TCOF1 expression when compared with cells transfected with non-targeting scrambled oligonucleotide. N=3 independent biological experiments. Data were deposited in NCBI GEO (acc. no. GSE299580). Table S4. The results of proteomics analysis of 786-O cells with silenced TCOF1 expression when compared with cells transfected with non-targeting scrambled oligonucleotide. N=4 independent biological experiments. Data have been deposited to the ProteomeXchange Consortium via the PRIDE partner repository with the dataset identifier PXD027601. Figure S1. TCOF1 silencing with double siRNA transfection results in more than 50% suppression of TCOF1 expression. The plot shows TCOF1 mRNA expression (% of control) in 786-O cells transfected with siRNA1, siRNA2 and or sequential transfection by siRNA1 followed by siRNA2. mRNA was isolated 48h after transfection, each bar represents TCOF1 expression in n = 3 wells of cell culture plate. Control: 786-O cells transfected with non-targeting scrambled oligonucleotide. Catalogue numbers and IDs of siRNAs are given in Methods. Figure S2. Stable expression of the RNA8S1 reference gene in cells with silenced TCOF1 expression. N=3 independent biological experiments. Statistical analysis was performed using t-test. P < 0.05 was considered statistically significant. Figure S3. Uncropped full scans of WB analysis of TCOF1 silencing in 786-O and Caki-1 cells. Each lane represents analysis of protein extract isolated from independently transfected cell culture flask. TCOF1 migrates at a higher apparent molecular weight than predicted based on its sequence, [file 12964_2026_2796_MOESM1_ESM.zip › Supplementary Data revised/Supplementary Figure S4_MTT_revised.docx]

**
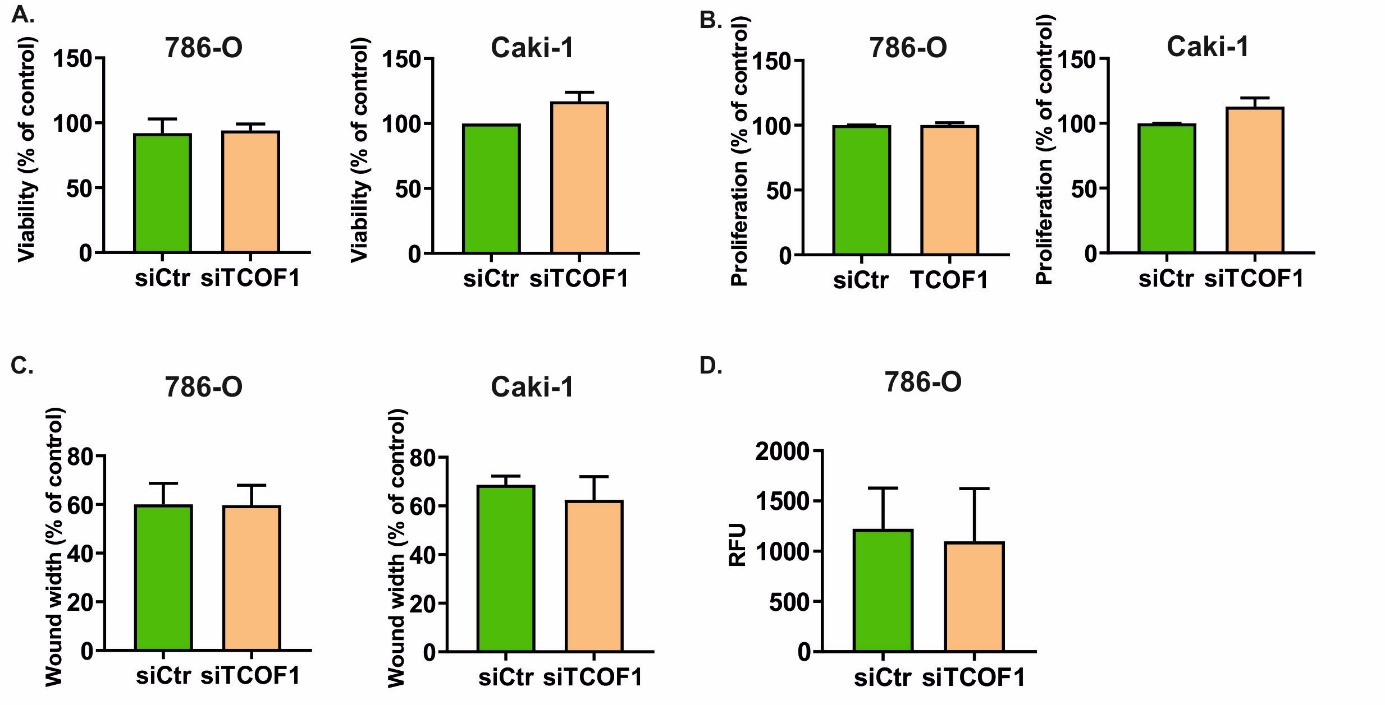
**

**Supplementary Figure S4. TCOF1 silencing does not affect proliferation, viability, migration, and adhesion-independent growth of ccRCC cells. A.** Viability. **B.** Proliferation. **C.** Migration. **D.** Adhesion-independent growth. 786-O and Caki-1 cells were transfected with siRNA targeting TCOF1 (siTCOF1) or non-targeting control scrambled oligonucleotide. The number of independent biological experiments: N=3 (panels A, B, C) n=4 (panel D). RFU=relative fluorescence units. Statistical analysis was performed using paired t-test. There were no statically significant changes.
